# Efficacy of combined Kinesio-taping with chest physiotherapy program on pain, pulmonary function, respiratory muscle strength and quality of life after mastectomy: a randomized controlled trial

**DOI:** 10.3389/fmed.2025.1664210

**Published:** 2026-01-22

**Authors:** Dalia Mahmoud Abdelmonem Elsherbini, Hadaya Mosaad Eladl, Nesma M. Allam, Moaz Abulfaraj, Mohamed El-Sherbiny, Nermine Nosseir, Ashraf Maghrabi, Wisam Jamal, Mohamed Mahmoud Abdelfattah Abdelrahman, Mohamed A. Eladl

**Affiliations:** 1 Department of Clinical Laboratory Sciences, College of Applied Medical Sciences, Jouf University, Sakaka, Saudi Arabia; 2 Department of Physical Therapy and Health Rehabilitation, College of Applied Medical Sciences, Jouf University, Sakaka, Saudi Arabia; 3 Department of Surgery, Faculty of Medicine, King Abdulaziz University, Jeddah, Saudi Arabia; 4 Department of Basic Medical Sciences, College of Medicine, AlMaarefa University, Riyadh, Saudi Arabia; 5 Research Center, Deanship of Scientific Research and Post-Graduate Studies, AlMaarefa University, Riyadh, Saudi Arabia; 6 Department of Biomedical Sciences, College of Medicine, Gulf Medical University, Ajman, United Arab Emirates; 7 Thoracic Surgery Division, Department of Surgery, Faculty of Medicine, King Abdulaziz University, Jeddah, Saudi Arabia; 8 Department of Surgery, Faculty of Medicine, University of Jeddah, Jeddah, Saudi Arabia; 9 Department of Anesthesia, Surgical Intensive Care and Pain Management, Faculty of Medicine, Mansoura University, Mansoura, Egypt; 10 Department of Anesthesia and Critical Care, King Abdulaziz University Hospital, King Abdulaziz University, Jeddah, Saudi Arabia; 11 Department of Basic Medical Sciences, College of Medicine, University of Sharjah, Sharjah, United Arab Emirates

**Keywords:** Kinesio-taping, mastectomy, pain, pulmonary function, quality of life, respiratory muscle strength

## Abstract

**Objectives:**

This study aimed to assess the effects of incorporating Kinesio-taping (KT) into a chest physical therapy program on alleviating pain and enhancing pulmonary function, respiratory muscle strength, and quality of life following mastectomy.

**Design:**

This was a prospective, randomized controlled trial.

**Setting:**

Physiotherapy outpatient clinic.

**Participants:**

Sixty participants aged 30–50 years who had undergone modified radical mastectomy were randomly allocated to either the Kinesio-taping or traditional physical therapy exercise group.

**Intervention:**

The Kinesio-taping group received both Kinesio-taping and traditional chest physiotherapy. The control group received only the traditional chest physiotherapy program. The interventions were administered over a 4-week period.

**Outcome measures:**

The primary outcome measure was pain, which was evaluated using a visual analogue scale (VAS). Secondary outcomes included pulmonary function, specifically forced vital capacity (FVC) and forced expiratory volume in one second (FEV1), assessed via spirometry; respiratory muscle strength, determined by measuring maximal inspiratory pressure (MIP) and maximal expiratory pressure (MEP) using a portable pressure device; and quality of life, assessed using the Short Form-36 (SF-36) questionnaire. All outcomes were measured at baseline and 4 weeks after the intervention.

**Results:**

The evaluated parameters exhibited significant alterations between the pre- and post-intervention after a duration of 4 weeks in both groups. The results indicated a significant changes (*p* < 0.001) in post-intervention measures compared to pre-intervention measures in both groups. No significant differences were observed between the groups before the intervention. The KT group demonstrated superior postintervention outcomes. Specifically, the KT group showed a significant reduction in VAS scores post-intervention than the control group (3.97 ± 1.65 vs. 6.50 ± 1.57, *p* < 0.001). Post-intervention, the KT group exhibited significantly higher values (*p* < 0.001) of FVC, FEV1, and FEV1/FVC (3.52 ± 0.64 L, 2.95 ± 0.56 L, and 85.09 ± 12.07%) compared to the control group (2.38 ± 0.56 L, 1.74 ± 0.54 L, and 72.39 ± 10.82%), respectively. Additionally, post-intervention MIP and MEP values were significantly higher (*p* < 0.001) in the KT group.

**Conclusion:**

Integrating Kinesio-taping into a standard chest physiotherapy program significantly reduced pain and enhanced pulmonary function, respiratory muscle strength, and quality of life in post-mastectomy patients compared to the application of traditional chest physiotherapy alone.

**Clinical trial registration:**

ClinicalTrials.gov, identifier NCT06701591.

## 1 Introduction

Modified radical mastectomy (MRM) is a comprehensive surgical procedure that involves the excision of breast tissue along with the axillary lymph nodes while preserving the underlying pectoral muscles. The procedure typically employs an oblique elliptical incision, which generally extends toward the axilla (1). In the postoperative phase, several factors, including pain and the respiratory depressant effects of general anesthesia, can adversely affect respiratory function. Pain, in particular, may restrict thoracic cage movement, thereby inhibiting respiratory muscle function (2).

Individuals who undergo modified radical mastectomy for breast cancer benefit significantly from timely and appropriate physical rehabilitation. Cardiovascular and respiratory complications are commonly observed, rendering them critical areas for postsurgical intervention in patients with breast cancer (3–5). Effective rehabilitation plays a crucial role in the recovery of physical health and function, leading to substantial improvements in quality of life (6).

The primary objective of early mobilization and postoperative respiratory therapy is to enhance respiratory function by improving thoracic mobility and facilitating the clearance of secretions (7). Conventional pulmonary rehabilitation typically involves physical exercise, training of inspiratory and expiratory muscles, airway clearance techniques, and various breathing exercises (8, 9).

Kinesio-taping (KT) involves the application of an elastic cotton tape coated with a hypoallergenic adhesive that can extend up to 140% of its original length. The therapeutic advantages of KT are believed to arise from a combination of biomechanical effects, sensory (exteroceptive) input, improved blood and lymphatic circulation, and analgesic mechanisms (10). Kinesiologic taping is currently employed for various musculoskeletal disorders, including impingement syndrome (11), shoulder pain (12), myofascial pain syndrome (13), sports injuries (14), and knee osteoarthritis (15).

Kinesio-taping is used as a complementary method alongside conventional therapeutic treatments to address postsurgical and respiratory issues, among other emerging evidence-based interventions in patient care. KT is suggested to alleviate pain by stimulating somatosensory receptors and creating a subtle lifting effect on the skin. In particular, during application, the skin must be manually stretched so that the elastic tape produces skin folds or convolutions. This lifting of the skin is hypothesized to expand the interstitial space, thereby facilitating lymphatic flow and drainage (8, 16). Additionally, when applied to the thoracic region, Kinesio-taping (KT) has demonstrated significant effectiveness in enhancing respiratory muscle performance and overall functional capacity in individuals with chronic obstructive pulmonary disease (COPD), contributing to improved lung function (9, 17, 18).

Previous studies have explored the effects of KT on postoperative pain in various patient groups, including those undergoing lobectomy (19), open-heart surgery (20), thoracotomy (21, 22), total knee replacement surgery (23), and laparoscopic cholecystectomy (24). Additionally, studies have investigated its impact on respiratory muscle strength and pulmonary function in patients with COPD (9, 25). However, there is a lack of research focusing on the effects of this intervention on pain, pulmonary function, respiratory muscle strength, and quality of life after mastectomy. Therefore, this study aimed to evaluate the influence of KT on these parameters in patients who underwent mastectomy.

## 2 Materials and methods

### 2.1 Study design

This was a prospective, single-blind, randomized controlled trial. This study was conducted in accordance with the ethical principles outlined in the Declaration of Helsinki and received approval from the Permanent Committee of Scientific Research Ethics at Jouf University (Approval No. 6905). Prior to initiation, the trial was registered at ClinicalTrials.gov (NCT06701591) and conducted between December 2024 and May 2025 at the Physical Therapy Outpatient Clinic of King Abdulaziz Hospital in Sakaka, Aljouf, Saudi Arabia. Participants were thoroughly informed about the study’s objectives and procedures, including their right to withdraw at any time. Written informed consent was obtained from all participants prior to enrolment. This study adhered to the CONSORT guidelines.

### 2.2 Participants

**Figure 1:**
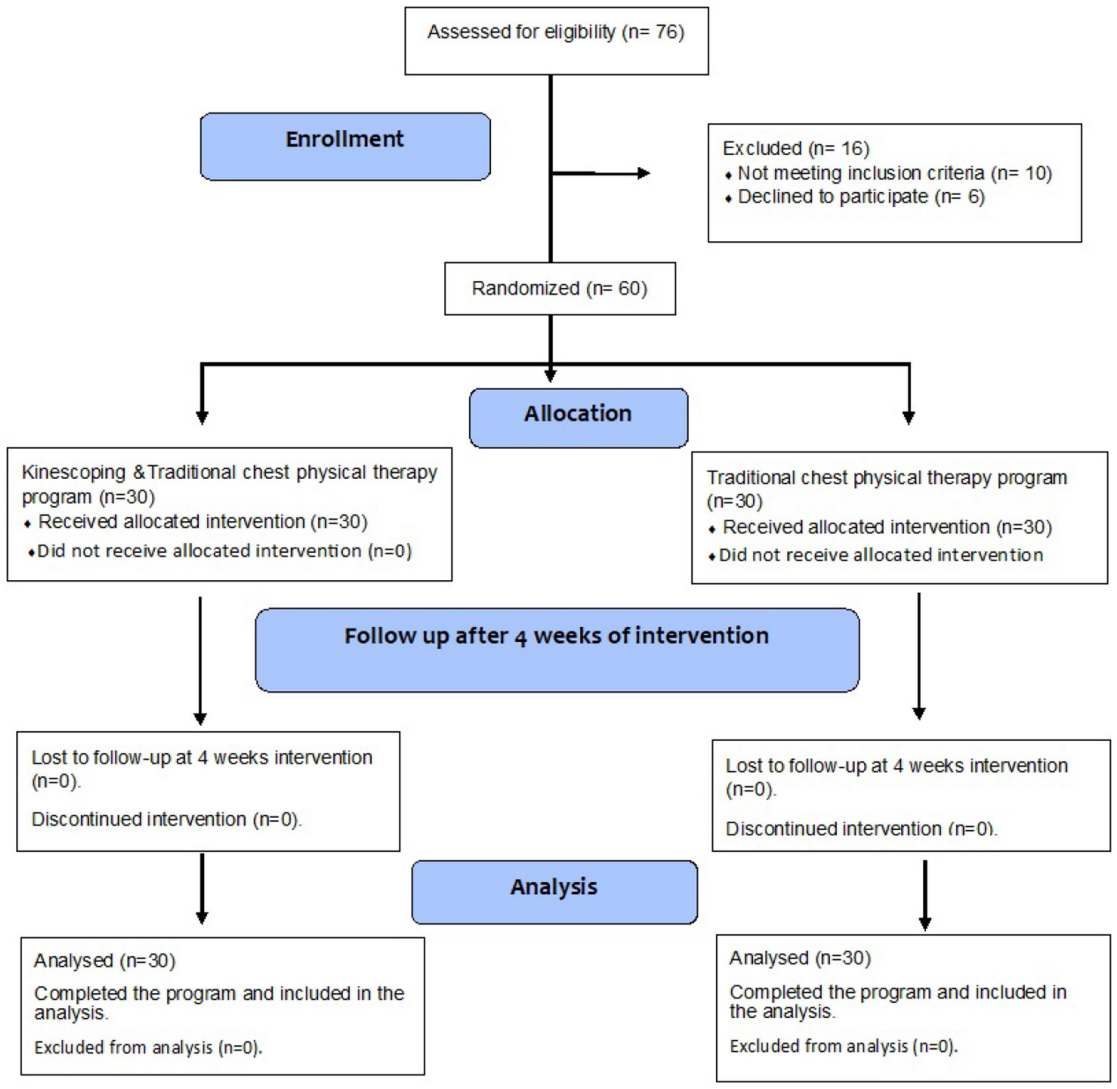
Consolidated standards of reporting trials (CONSORT) flowchart of the study.

The third author, who was blinded to both the intervention and outcome assessments, evaluated the participants’ eligibility based on the inclusion and exclusion criteria. To mitigate bias, the researcher did not participate in the administration of the intervention or outcome evaluations. Seventy-six participants were enrolled in this trial. Sixteen were excluded: 10 did not meet the inclusion criteria, and six declined to participate (Figure 1). The inclusion criteria comprised females aged 30–50 years who had undergone modified radical mastectomy within 10–14 days (after complete healing of the incision), experienced a pain level greater than 3 on the visual analogue scale (VAS), had a body mass index (BMI) below 30 kg/m^2^, and were able to understand instructions, as well as willing and cooperative to participate. Participants were excluded if they had sensitive skin, scars, lesions, or unhealed incisions at the application site; uncontrolled diabetes; deep vein thrombosis; prior chest diseases; congestive heart failure; pregnancy; central or peripheral nervous system disorders; altered sensations; active infections; required ventilation for more than 12 h; or had unstable hemodynamics. All participants received a brief explanation and demonstration of the testing procedure. Sixty participants were randomly assigned to one of two groups: Group A (KT group, *n* = 30), which received kinesio-taping combined with a chest physiotherapy program three times a week for 4 weeks, and Group B (control group, *n* = 30), which received only the chest physiotherapy program three times a week for 4 weeks.

### 2.3 Sample size calculation

The sample size was determined using G*Power software for Windows (version 3.1.9.7) according to the specified procedures (26). This calculation was based on prior studies (18, 21). The postoperative mean values for the Kinesio-taping and control groups were 4.65 and 6.57 in the first study, and 1.75 and 4.67 for the visual analogue scale (VAS) in the second study. The standard deviations (SD) within the groups were 1.79 and 1.74 for the first study and 1.41 and 1.68 for the second, resulting in effect sizes (*d*) of 1.09 and 1.88, respectively. Based on these calculations, the required total sample sizes to achieve 95% power at a 5% significance level were 46 and 18, respectively. Utilizing a smaller effect size of 1.09, a total sample size of 46 participants divided into two groups was selected. To account for a potential dropout rate of up to 25%, 30 participants per group were selected to ensure statistical adequacy.

### 2.4 Randomization

Sixty participants who had undergone modified radical mastectomy were randomly assigned to the KT or control group. To minimize bias and ensure group equivalence, block randomization with a 1:1 allocation ratio was utilized via http://www.randomization.com/, employing block sizes of 4, 6, and 8. The randomization process was conducted by the first author, who was not involved in participant recruitment, intervention delivery, or outcome assessment. To maintain allocation concealment, the randomization codes were sequentially numbered and placed in opaque, sealed envelopes for secure storage.

### 2.5 Intervention

In this study, the KT group received applications of Kinesio® Tex Gold™ (Kinesio Holding Corp., Albuquerque, NM, USA), an elastic, 100% cotton, and latex-free tape with a width of 5 cm. A single certified and KT-trained physical therapist applied the tape while the participants were seated. The tape was positioned bilaterally at the end of expiration, transversely across the fifth to sixth and ninth to tenth intercostal spaces, parallel to the incision, and parallel to the diaphragm at the level of the epigastric angle, using 50% stretch tension. The facilitation technique, designed to enhance muscle function, was employed; this method involves taping with a stretch of 25–50% as recommended (9, 27). Kinesio-taping was not applied directly over the incision but was placed parallel to the surgical site only after confirming that the wound was non-infected and intact. The KT was replaced every 5 days, for a total of six applications, by the same author. The participants were instructed to maintain the tape continuously but were permitted to shower with it. Post-treatment evaluations were conducted after 4 weeks and recorded by the second author, who was blinded to the treatment groups (18).

#### 2.5.1 Traditional chest physical therapy program

All participants in the KT and control groups received a chest physical therapy program, which consisted of deep breathing exercises, modified postural drainage, and supported cough, three times a week for 4 weeks.

#### 2.5.2 Deep breathing exercises

The researcher responsible for conducting the program sessions demonstrated proper diaphragmatic breathing using a series of exercises. Following the demonstration, the participants participated in a guided deep breathing routine characterized by a controlled exchange of breaths. This practice was performed in three sets, each consisting of 10 repetitions (17, 28).

##### 2.5.2.1 Deep diaphragmatic breathing

The exercise was performed with the patient seated upright, with support provided to the back, and the pelvis positioned in a posterior tilt. This alignment improves the diaphragm’s length-tension relationship, thereby supporting more efficient breathing. The patient was guided to keep her upper chest and shoulders relaxed and breathe in deeply through the nose, focusing on expanding the lower ribcage and abdomen to create a balloon-like rise of the belly, after which the patient exhaled smoothly. If there is any trouble relaxing the accessory respiratory muscles, a contract-relax technique can be applied beforehand to help promote muscle relaxation (29).

##### 2.5.2.2 Thoracic expansion exercises (TEEs)

This particular deep breathing exercise focuses on the inhalation phase, which is an active process, and frequently includes a brief 3-s breath hold at the peak of inspiration. The exercises are designed to engage specific lung regions, including the apical, upper lateral, middle, lower lateral, and posterior basal areas, and can be performed unilaterally (on one side of the chest) or bilaterally (on both sides) (30). Unilateral thoracic expansion exercises (TEEs) are particularly advantageous for patients who have undergone postoperative thoracic surgery. The optimal position for performing these exercises is side-lying, with the operated side facing upward and the affected arm abducted and elevated to head level (29). Initially, the researcher supported the incision site by consistently applying gentle pressure with their hand. Placing the hand over the surgical area also provides tactile stimulation that can facilitate lung expansion (31). The participant was then instructed to breathe in deeply and slowly, with the aim of raising the physiotherapist’s hand during inhalation, and to hold their breath for 2–3 s before a slow exhalation through the mouth. Typically, approximately three repetitions of the expansion exercise were considered sufficient. Furthermore, supporting the chest drain site during thoracic expansion exercises is essential for minimizing pain and enabling the patient to take deep breaths comfortably (31).

##### 2.5.2.3 Supported cough

The cough support technique was implemented after the patient took several deep breaths. To facilitate effective coughing, the patient was instructed to elevate their chin, support the surgical site with a pillow, and inhale deeply while extending their body. The patient briefly held their breath, and with continuous support over the surgical area, executed a series of forceful exhalations while leaning forward to increase intra-abdominal pressure (29, 32).

##### 2.5.2.4 Modified postural drainage positions

Modified postural drainage positions are the most commonly used in postoperative patients who cannot tolerate traditional head-down postures due to risks such as aspiration, vomiting, shortness of breath, cardiovascular instability, or reduced oxygen levels. Suggested modifications include using alternative positions, such as the horizontal lateral decubitus or supine position, to closely mimic the traditional posture (33), or adopting a forward-leaning posture while seated (31). Modified postural drainage positions were employed independently or in combination with other airway clearance techniques. To ensure comprehensive treatment of all affected pulmonary regions, multiple positions were utilized during each session, with each position maintained for 5–10 min or as long as the patient could tolerate. The patient’s face was consistently kept visible to monitor for any signs of discomfort or intolerance throughout the procedure. The procedure was immediately discontinued if the patient exhibited any symptoms, such as difficulty breathing, cyanosis, hemoptysis, dizziness, nausea, vomiting, anxiety, tachycardia, bronchospasm, elevated blood pressure, or any discomfort or pain in the musculoskeletal system (29).

Applying chest vibrations during exhalation over the lung areas situated below the surgical incision is generally more comfortable than percussion and serves as an effective alternative technique (29, 34). Manual vibration was used with modified postural drainage positions to facilitate secretion removal. To execute the technique correctly, the physiotherapist positioned their palms flat on the patient’s chest, either side-by-side or with one hand placed over the other. Following deep inhalation by the patient, the physiotherapist applied gentle rhythmic oscillations to the chest wall throughout the entire exhalation phase (29).

### 2.6 Safety monitoring and adverse events

After complete healing from a modified radical mastectomy, kinesio-taping on the chest is generally safe; however, it requires careful monitoring of skin integrity, scar response, and sensation. The most important safety checks include watching for redness, itching, blistering, increased swelling, or discomfort, especially because post-mastectomy skin may be sensitive. Adverse events are usually mild and include skin irritation, dermatitis, blistering, or temporary swelling if the tape is applied too tightly or in the wrong direction; more serious issues, such as lymphatic congestion, are rare. To ensure safety, tape was applied with suitable tension, removed gently, avoided over fragile or irradiated skin, and worn only for short periods, with the skin resting between applications (35).

### 2.7 Outcome measures

The primary outcome of the study was pain, which was measured using the visual analogue scale (VAS). Secondary outcomes included pulmonary function, assessed using a spirometer; respiratory muscle strength, evaluated using a portable pressure measurement device; and quality of life, measured using the Short Form-36 (SF-36) questionnaire. All outcomes were documented at the start of the study and after 4 weeks of intervention.

Prior to the assessment, all participants were provided with a brief, standardized explanation detailing the purpose of each test, the procedural steps involved, and the expectations for participant conduct during the procedure. This verbal briefing ensured that each participant comprehensively understood the sequence of measurements, requisite body positions, and signals employed to initiate or terminate each test. Following the verbal explanation, the researcher conducted a practical demonstration of each assessment technique, including the execution of respiratory function tests, pain scoring, and measurement of respiratory muscle strength. This demonstration enabled the participants to visually observe the correct performance, thereby reducing anxiety or uncertainty associated with unfamiliar procedures. Participants were then given the opportunity to ask questions, and clarifications were provided as necessary. This process ensured that all tests were conducted safely and consistently, with the participants possessing a full understanding of the procedures.

#### 2.7.1 Visual analogue scale (VAS)

A linear scale serves as a visual tool to represent the spectrum of pain that a patient thinks they are experiencing. Typically, this scale is 10 cm in length, with or without markings at each centimeter to illustrate the range. One extremity of the scale signifies the most severe pain the patient can imagine, while the opposite end denotes the absence of pain. The patient indicated their level of discomfort by marking a point on the scale. A score of 0 corresponds to no pain, a score above 5 indicates significant pain, and a score of 10 represents the maximum pain intensity (36).

#### 2.7.2 Pulmonary function tests

Pulmonary function was assessed using a spirometer (Pony FX; COSMED Inc., Rome, Italy). The parameters measured included forced vital capacity (FVC), the total volume of air that can be forcefully exhaled after a full inhalation, expressed in liters; forced expiratory volume in one second (FEV1), the amount of air expelled during the first second of a forceful exhalation, also in liters; and the FEV1/FVC ratio. All assessments were performed in accordance with the standards set by the American Thoracic Society (ATS) and the European Respiratory Society (ERS) (37). Prior to the spirometry testing, the equipment was calibrated, and each patient received standardized instructions on how to perform the test. During the procedure, the participants were seated on a chair with back support but no armrests, with their knees bent at a 90-degree angle. A nose clip was applied to close the nostrils and prevent air leakage between the mouth and mouthpiece (6, 38). The participant was instructed to take a rapid, maximal breath in starting from functional residual capacity while holding the breathing tube in place with lips tightly sealed around the mouthpiece and ensuring that the tongue did not obstruct airflow. This was followed by a strong exhalation, expelling as much air as possible over 3 s. Forced vital capacity (FVC) and forced expiratory volume in one second (FEV1) were determined from three valid forced exhalation attempts, each initiated properly and without errors. The highest FVC and FEV1 values obtained were recorded (6).

#### 2.7.3 Respiratory muscle strength

Respiratory muscle strength was evaluated by measuring maximal inspiratory pressure (MIP), indicative of the strength of the inhalation muscles, and maximal expiratory pressure (MEP), which reflects the strength of exhalation muscles. These measurements were obtained using a portable pressure measurement device (Pony FX; COSMED Inc., Rome, Italy) (22). The participants were seated and equipped with a nose clip throughout the assessment. To determine the maximal inspiratory pressure (MIP), the participants were instructed to inhale forcefully against the device’s mouthpiece. For maximal expiratory pressure (MEP), they performed maximal exhalation and sustained it for 1–2 s. Each test was conducted three times, and the highest values, expressed in cm H_2_O, were recorded for analysis (39).

#### 2.7.4 Quality of life

The quality of life (QoL) was evaluated utilizing the Short Form-36 (SF-36) questionnaire, which consists of 36 items categorized into eight domains: physical functioning, social functioning, role limitations due to physical health, general health perceptions, bodily pain, role limitations due to emotional problems, vitality, and mental health. Each domain is scored on a scale ranging from 0 to 100, with higher scores signifying superior overall well-being (22). The Arabic version of the SF-36 was used in this study because it has been validated for reliability and validity (40).

### 2.8 Statistical analysis

Data analysis was conducted using GraphPad Prism version 9, and the results are expressed as mean ± standard deviation (SD). Differences in demographic variables between the KT (experimental) and control groups were evaluated using the Student’s t-test. A two-way repeated-measures ANOVA was used to assess the outcome variables between groups over time. Categorical data are presented as percentages and were compared using the chi-square test. Partial eta squared was used to calculate the effect sizes between the groups. Statistical significance was set at *p* < 0.05. Within-group differences in outcomes were analyzed using paired t-tests. Outliers were excluded after data standardization. Furthermore, linear regression analysis was performed to investigate the relationship between quality of life—assessed via the SF-36 physical component summary (PCS)—and respiratory muscle strength variables (MIP and MEP), which showed significant correlations. Statistical significance was set at *p* < 0.05. All assumptions for the linear regression analysis were verified, including homoscedasticity (equal variance), linearity, normality, and the absence of multicollinearity among the independent variables. Prior to the application of parametric tests, normality and homoscedasticity of the variance were assessed.

## 3 Results

### 3.1 Subject characteristics

Table 1 outlines the participant characteristics of the KT and control groups. No significant differences were observed between the groups in terms of average age, height, weight, cancer stage, or type of treatment received (*p* > 0.05).

**Table 1:** Demographic characteristics of the participants.

Characteristics	Mean ± SD		MD	Effect size (*η*^2^)	*p*	95% CI	
	KT group (*N* = 30)	Control group (*N* = 30)				Lower	Upper
Age (years)	42.10 ± 6.59	43.08 ± 5.57	−0.98	0.007	0.37 (ns)	−4.14	2.17
Weight (Kg)	71.44 ± 5.13	71.79 ± 5.80	−0.35	0.001	0.80 (ns)	−3.18	2.48
Height (cm)	165.9 ± 4.59	164.6 ± 4.07	1.25	0.02	0.27 (ns)	−0.99	3.49
BMI (kg/m^2^)	26.02 ± 2.31	26.50 ± 1.98	−0.47	0.01	0.40 (ns)	−1.59	0.06
Length of hospital stay (days)	10.20 ± 2.83	11.03 ± 2.41	−0.83	0.03	0.23 (ns)	−2.19	0.53
						*Χ*^2^ (*p*-value)	
Cancer stages						0.287 (0.59)	
Stage II	18 (60%)	20 (66.7%)					
Stage III	12 (40%)	10 (33.3%)					
Modalities of treatment						0.067 (0.80)	
Radiotherapy	13 (43.3%)	14 (53.3%)					
Radiotherapy + Chemotherapy	17 (56.7%)	16 (53.3%)					

BMI, Body mass index, N, number.

Mean ± SD is used to provide quantitative data.

*p* ≥ 0.05 is statistically non-significant (ns).

Χ^2^: Chi-square test is used to provide categorical data, ˆ*p* value < 0.05 = statistically significant chi-square test.

### 3.2 Clinical measures

**Figure 2:**
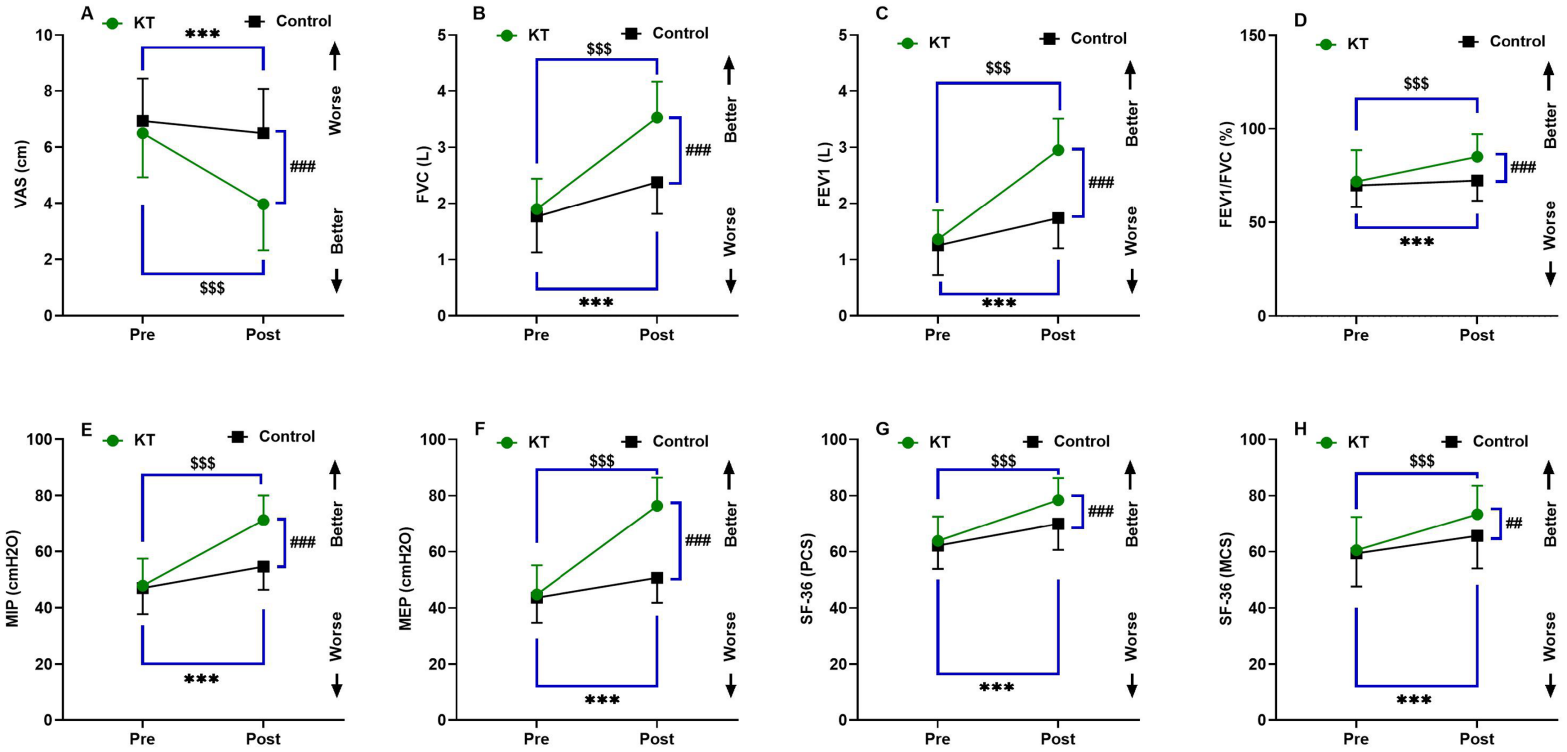
Interactions between group and time for **(A)** VAS, **(B)** FVC (L), **(C)** FVC1 (L), **(D)** FVC1/FVC (%), **(E)** MIP (cmH_2_O), **(F)** MEP (cmH_2_O), **(G)** SF-36 (PCS), **(H)** SF-36 (MCS). Data are expressed as the mean ± SD. ^***^p < 0.001 within control group pre *vs.* post intervention, ^$$$^*p* < 0.001 within KT group pre *vs.* post intervention. ^##^*p* < 0.01, ^###^*p* < 0.001 of KT *vs.* control group post-intervention. VAS, visual analog scale; FVC, forced vital capacity; FEV1, forced expiratory volume in 1 s; MIP, maximal voluntary inspiratory pressure; MEP, maximal expiratory pressure; SF-36 (PCS), Short Form 36 (SF-36) Health Survey Physical Component Summary; SF-36 (MCS), Short Form 36 (SF-36) Health Survey Mental Component Summary.

Table 2 and Figure 2 illustrate significant alterations in VAS scores from pre-intervention (6.50 ± 1.57 for the KT group and 6.93 ± 1.51 for the control group) to post-intervention (3.97 ± 1.65 for the KT group and 6.50 ± 1.57 for the control group). Furthermore, the KT group exhibited a significantly greater reduction in VAS scores post-intervention than the control group (*p* < 0.001).

**Table 2:** Comparison of the groups in terms of pre- and post-intervention clinical measures.

Variable	KT group				Control group				Group × Time interaction (*F*) *p* value	*p*^b^ Inter-group changes Post treatment	Effect size (*η*^2^)
	Pre	Post	MD (95%CI)	*p*^a^ Intra-group changes	Pre	Post	MD (95%CI)	*p*^a^ Intra-group changes			
VAS (cm)	6.50 ± 1.57	3.97 ± 1.65	−2.53 (−2.73 to −2.34)	<0.001	6.93 ± 1.51	6.50 ± 1.57	−0.43 (−0.65 to −0.22)	<0.001	*F*_1,58_ = 19.69	<0.001	0.39
									<0.001		
FVC (L)	1.90 ± 0.54	3.52 ± 0.64	1.63 (1.40–1.85)	<0.001	1.77 ± 0.64	2.38 ± 0.56	0.62 (0.46–0.77)	<0.001	*F*_1.58_ = 90.16	<0.001	0.48
									<0.001		
FEV1 (L)	1.36 ± 0.52	2.95 ± 0.56	1.59 (1.40–1.77)	<0.001	1.25 ± 0.53	1.74 ± 0.54	0.49 (0.38–0.60)	<0.001	*F*_1.58_ = 103.4 <0.001	<0.001	0.56
FEV1/FVC (%)	71.88 ± 16.78	85.09 ± 12.07	13.21 (9.55–16.86)	<0.001	69.81 ± 11.65	72.39 ± 10.82	2.58 (1.83–3.33)	<0.001	*F*_1.58_ = 11.97	<0.001	0.24
									<0.01		
MIP (cmH_2_O)	47.88 ± 9.58	71.26 ± 8.81	23.37 (20.99–25.75)	<0.001	47.05 ± 9.29	54.65 ± 8.25	7.60 (5.67–9.52)	<0.001	*F*_1.58_ = 79.93	<0.001	0.49
									<0.001		
MEP (cmH_2_O)	44.79 ± 1.35	76.43 ± 10.02	31.64 (29.70–33.58)	<0.001	43.62 ± 8.88	50.73 ± 8.90	7.11 (5.98–8.24)	<0.001	*F*_1.58_ = 108.9	<0.001	0.66
									<0.001		
SF-36 (PCS)	63.87 ± 6.68	78.43 ± 7.84	14.57 (12.14–17.00)	<0.001	62.20 ± 8.32	69.97 ± 9.34	7.77 (6.11–9.42)	<0.001	*F*_1.58_ = 57.91	<0.001	0.20
									<0.001		
SF-36 (MCS)	60.53 ± 11.87	73.37 ± 10.24	12.83 (10.96–14.71)	<0.001	59.40 ± 11.81	65.67 ± 11.64	6.27 (4.71–7.82)	<0.001	*F*_1.58_ = 26.60	<0.001	0.11
									<0.001		

Values are shown as mean ± standard deviation (SD).

VAS, Visual Analog Scale; FVC, Forced vital capacity; FEV1, Forced expiratory volume in 1 s; MIP, Maximal voluntary inspiratory pressure; MEP, Maximal expiratory pressure; SF-36 (PCS), Short Form 36 (SF-36) Health Survey Physical Component Summary; SF-36 (MCS), Short Form 36 (SF-36) Health Survey Mental Component Summary; MD, Mean Difference; CI, Confidence Interval.

*p*^a^-value Intra-group changes from Paired-Samples T Test.

*p*^b^-value Inter-group changes between KT and control group post intervention from Independent-Samples T Test.

*η*^2^: partial eta square between KT and control group post intervention.

Group × time interaction *p* value from two-way repeated measures ANOVA.

Comprehensive pulmonary function tests were conducted, revealing a significant increase in FVC, FEV1, and FEV1/FVC values (*p* < 0.001) following the intervention in both the KT and control groups. Prior to the intervention, no significant differences were observed between the groups for these variables (*p* < 0.05). However, post-intervention, the KT group demonstrated significantly higher values of FVC, FEV1, and FEV1/FVC (3.52 ± 0.64 L, 2.95 ± 0.56 L, and 85.09 ± 12.07%, respectively) compared to the control group (2.38 ± 0.56 L, 1.74 ± 0.54 L, and 72.39 ± 10.82%, respectively).

Additionally, respiratory muscle strength assessments indicated significant increases in MIP and MEP values (*p* < 0.001) post-intervention in both groups. No significant differences were noted between the groups for these measures before the intervention (*p* < 0.05). Post-intervention, the KT group exhibited significantly higher (*p* < 0.001) MIP and MEP values (71.26 ± 8.81 and 76.43 ± 10.02 cmH_2_O, respectively) than the control group (54.65 ± 8.25 and 50.73 ± 8.90 cmH_2_O, respectively).

With respect to HRQoL, the SF-36 physical component summary (PCS) and mental component summary (MCS) scores showed significant improvements (*p* < 0.001) following the intervention in both the KT and control groups. There were no significant differences in these scores between the groups before the intervention (*p* > 0.05). However, post-intervention, the KT group had significantly higher SF-36 PCS and MCS scores (78.43 ± 7.84 and 73.37 ± 10.24) compared to the control group (69.97 ± 9.34 and 65.67 ± 11.64), with *p*-values of <0.001 and <0.01, respectively.

Two-way repeated measures ANOVA showed significant changes in VAS, FVC, FEV1, FEV1/FVC, MIP, MEP, and SF-36 (PCS) between the KT and control groups post-intervention, as evidenced by an effect size (*η*^2^) > 0.14.

### 3.3 Adverse effects

Within the KT Group, two patients experienced mild adverse effects associated with the application of KT, specifically minor skin irritation, and temporary discomfort. These reactions were mild, resolved promptly, and did not need treatment continuation. Appropriate interventions, such as removing the tape, allowing the skin to rest, and adjusting the taping technique, were sufficient to manage the symptoms, and no further complications were observed.

### 3.4 Correlation between MIP, MEP and SF-36 (PCS)

**Figure 3:**
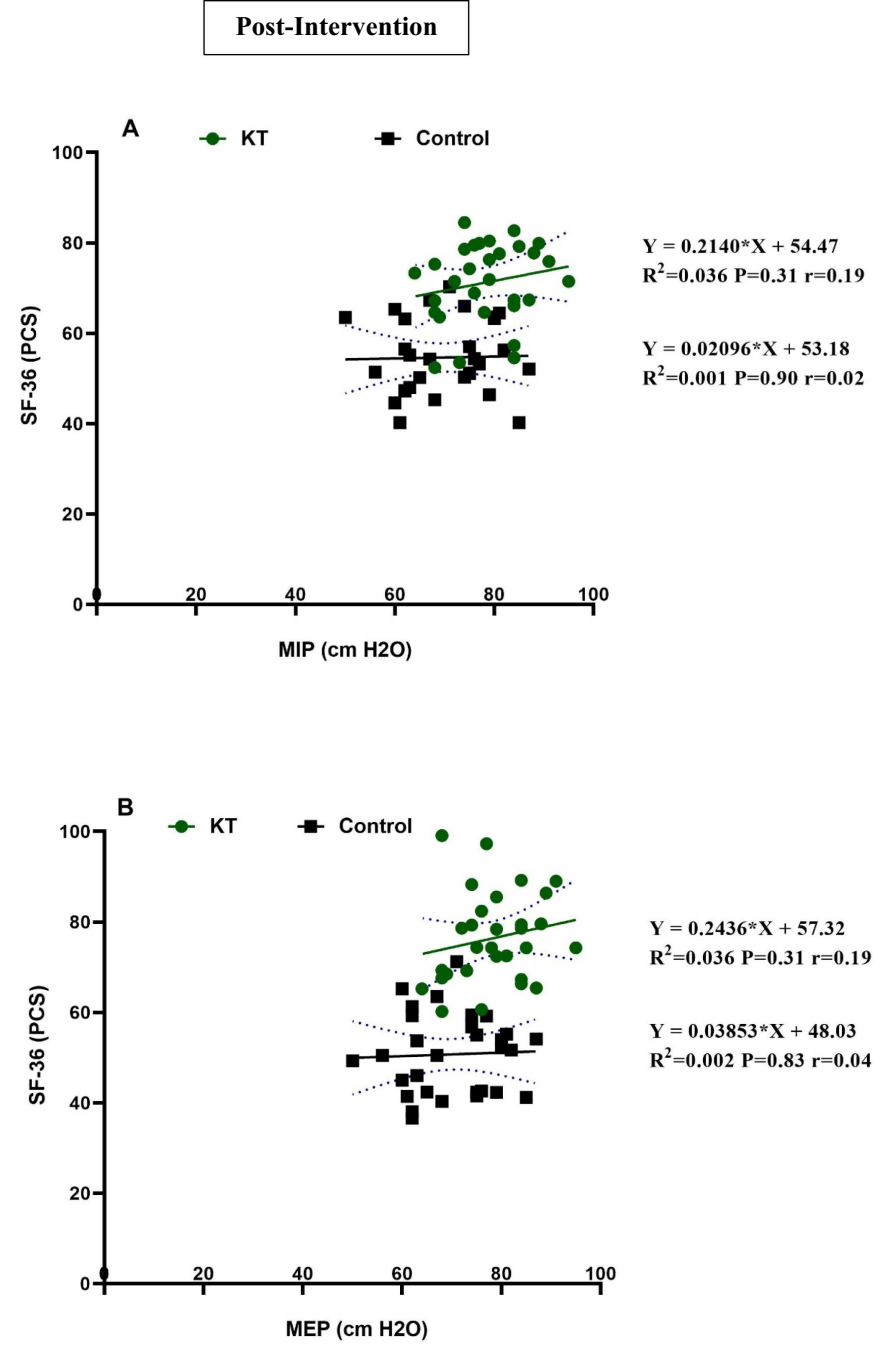
Linear regression of the following parameters post-intervention: **(A)** MIP and SF-36 (PCS) and **(B)** MEP and SF-36 (PCS). MIP, Maximal voluntary inspiratory pressure; MEP, Maximal expiratory pressure; SF-36 (PCS), Short Form 36 (SF-36) Health Survey Physical Component Summary.

Table 3 presents a weak positive correlation between maximal inspiratory pressure (MIP) and the SF-36 physical component summary (PCS) in both the KT (*r* = 0.19) and control groups (*r* = 0.02). Linear regression analysis did not yield statistically significant results. In the KT group, the enhancement in MIP accounted for a 3.6% increase in the SF-36 (PCS) score, whereas in the control group, it resulted in only a 0.1% increase (Figure 3A). A similarly weak positive correlation was observed between maximal expiratory pressure (MEP) and SF-36 (PCS) in both groups, with correlation coefficients of *r* = 0.19 in the KT group and *r* = 0.04 in the control group. Linear regression analysis of MEP also did not reveal statistically significant outcomes. In the KT group, the increase in MEP was associated with a 3.6% improvement in the SF-36 (PCS), compared to a 0.2% increase in the control group following the intervention (Figure 3B).

**Table 3:** Factors associated with SF-36 (PCS) in KT group *vs*. control T group post intervention.

Regression statistics	MIP (cm H_2_O)		MEP (cm H_2_O)	
	KT group	Control group	KT group	Control group
Regression parameters				
*B* (95% CI)	0.17 (−0.17, 0.51)	0.03 (−0.41, 0.47)	0.15 (−0.15, 0.45)	0.04 (−36, 0.45)
Beta	0.19	0.02	0.19	0.04
SE	0.17	0.21	0.15	0.20
*t*-value	1.03	0.13	1.028	0.21
*p*-value	0.31 (ns)	0.90 (ns)	0.31 (ns)	0.83 (ns)
Correlation				
Correlation coefficient (*r*)	0.19	0.02	0.19	0.04
*R* ^2^	0.036	0.001	0.036	0.002
Adjusted *R*^2^	0.002	−0.035	0.002	−0.034

*B*: unstandardized coefficient estimate.

Beta: standardized coefficients estimate.

SE: standard error of the coefficient estimate.

CI, confidence interval.

*p* ≤ 0.05 is statistically significant, ns: non-significant.

*r*: Pearson coefficient.

*R*^2^: R square coefficient of determination.

## 4 Discussion

The primary findings of this study indicate that Kinesio-taping (KT) significantly reduced pain levels (*p* < 0.001). Furthermore, patients who underwent mastectomy demonstrated notable improvements in pulmonary function, respiratory muscle strength, and QoL (*p* < 0.001). The postoperative effects of general anesthesia may impair the diaphragm and other inspiratory muscles, leading to altered rib cage movement during respiration and a reduction in lung volume (41). Post-surgery, respiratory muscle strength and reserve decrease due to compromised respiratory function. Additionally, postoperative pain restricts diaphragmatic motion by inhibiting the phrenic nerve, resulting in decreased diaphragmatic strength (42). Consequently, breathing becomes shallower, leading to reduced lung volume and capacity. The cough reflex is weakened, resulting in mucus retention, which can contribute to atelectasis, hypoxemia, hypercapnia, and respiratory failure (43). Postoperative pulmonary complications extend patients’ stays in both the intensive care unit and hospital, thereby increasing healthcare expenditures (44). In the initial hospital phase following modified radical mastectomy (MRM), pulmonary rehabilitation is crucial for mitigating the risk of respiratory complications (45). Therefore, this study aimed to assess the impact of integrating Kinesio-Taping (KT) with a chest physical therapy program on pain management, pulmonary function, respiratory muscle strength, and quality of life following MRM. The effects of Kinesio-Taping (KT) vary according to the degree of stretch applied and the site of application. These variations have led to the development of diverse techniques. For example, muscle techniques are employed to stimulate or relax specific muscles. In this study, a stimulation technique was used to enhance respiratory muscle function, following the protocol suggested by Kase (27).

Our findings indicate a reduction in pain following the application of KT in the experimental group compared to that in the control group (*p* < 0.001). The beneficial effect of KT on postoperative pain may be attributed to the tension it induces in soft tissue structures, potentially facilitating smoother and less painful movements. Furthermore, KT may modulate pain through the gate control theory by enhancing neuromuscular signaling via afferent feedback. By increasing sensory input to large-diameter nerve fibers, KT may alleviate pain perception by diminishing the signals from smaller nerve fibers that transmit pain impulses (20). KT may also assist in re-educating the neuromuscular system, alleviating pain, reducing inflammation, enhancing performance, and promoting circulation, which facilitates lymphatic drainage and reduces inflammation and edema (46, 47). Another proposed mechanism is that KT reduces pain by stimulating the sensory pathways involved in interconnected, multimodal, and cross-modal neural networks (48). Keratinocytes may function as non-neural primary detectors of the mechanical stimuli produced by KT, potentially activating nearby C fibers through a signal transduction process, such as intracellular calcium flux (Ca^2+^) (49). Our results are consistent with those of previous studies on various thoracic surgeries (19, 21, 22, 50, 51). KT following lobectomy for lung cancer has been demonstrated to be a safe and effective adjunctive treatment for managing chest pain. It enhances the efficacy of oral pain medications, as evidenced by a significant reduction in pain scores on the Visual Analogue Scale (VAS) observed on postoperative days 5, 8, and 30 (19). Similarly, KT is a reliable and straightforward technique for managing postoperative pain in patients undergoing thoracotomies. When applied early, it may help prevent the onset of postoperative complications (21, 22). Moreover, KT is a safe, drug-free, and cost-effective approach that shows promise in reducing postoperative pain, decreasing the need for analgesic medications, and consequently minimizing the risk of side effects associated with these drugs following sternotomy surgery (50, 51). Studies involving laparoscopic cholecystectomy have demonstrated that KT significantly lowers abdominal pain perception as early as the first postoperative day. Additionally, it improved effort tolerance and reduced the need for pain relief medications compared with the control group (24, 52).

In contrast to our findings, previous research has indicated that the integration of KT into chest physiotherapy does not result in a significantly greater reduction in pain compared to chest physiotherapy alone following open heart surgery (20). This discrepancy may be attributed to several factors, including the limited duration of KT application (restricted to 7 days), differences in incision sites, variations in application techniques, small sample sizes, and variations in physiological factors that may influence patient cooperation during the study. Similarly, KT did not provide any additional benefit in terms of pain reduction or enhancement of quality of life in participants treated with non-steroidal anti-inflammatory drugs (NSAIDs) for post-thoracotomy pain syndrome (53). These conflicting results may be attributed to the short duration of KT application, which lasted only 5 days, as well as significant differences in the duration of pain and patient age at baseline between the treatment groups or differing application techniques.

The study found that The KT group exhibited better lung function than the control group (*p* < 0.001). This improvement might be due to more space for air and better lymph flow, which helped the chest move and improved breathing with the KT (18). Thoracic KT helps with breathing out by placing tape around the chest, following the natural movement of the chest when breathing in. As the person breathes out, the tape provides a balancing effect (17, 27). The characteristics of the tape may have contributed to enhancements in pulmonary function. These findings are consistent with previous research conducted in different groups KT is both an effective and safe intervention for enhancing pulmonary function following thoracotomy (17, 21, 54, 55). It should be integrated into post-thoracotomy pulmonary rehabilitation programs (21, 22). Additionally, KT facilitates respiratory function post-sternotomy (50, 51). The application of taping to the intercostal muscles has demonstrated significant improvements in patients with COPD, enhancing lung function metrics such as FEV1 and FEV1/FVC, as well as exercise capacity. Therefore, thoracic KT, along with regular physiotherapy, can lead to better patient outcomes (17, 18, 56). In athletes, KT has been shown to improve breathing volume during exercise while maintaining a steady breathing rate (54). Using KT on the diaphragm muscle improves aerobic capacity and short-term lung function, especially FEV1 and the FEV1/FVC ratio, compared to a fake KT group in inactive adults (55).

In contrast, applying KT to the diaphragm and scalene muscles in patients with COPD did not result in any statistically significant changes in spirometric measurements, such as FEV1 and PEF (57). These negative results may be due to the fact that only a single KT session was applied, as the authors suggested that one session alone may be insufficient to produce a meaningful improvement in pulmonary function, different techniques of application, and population. Similarly, KT cannot significantly improve pulmonary function variables (FEV1 and FVC) when used alongside chest physiotherapy, as opposed to chest physiotherapy alone after open-heart surgery (20). This contradiction might be due to the short duration of KT (7 days only), different technique of application, incision site, small sample size, and variability in physiological parameters, which may also affect the level of patient cooperation.

The results of the current study showed enhanced respiratory muscle strength after KT application in the study group compared to the control group (*p* < 0.001). The mechanism by which KT enhances respiratory muscle strength involves stimulating the sensory-motor and proprioceptive systems to facilitate muscle activity, assisting weakened muscles in regaining normal function, addressing misalignments by reducing muscle spasms, and facilitating the drainage of lymphatic fluid or subcutaneous bleeding by enhancing local blood flow (27, 58–60).

Consistent with the findings of this study, earlier research has reported a significant impact of KT on respiratory muscular strength (22, 50, 51, 57). Applying KT enhances respiratory muscle strength during the postoperative period after thoracotomy. Consequently, KT is considered a valuable addition to pulmonary rehabilitation programs after thoracotomy (22). Similarly, KT enhances inspiratory muscle strength following sternotomy (50, 51). Additionally, a single day of KT application significantly improved MIP and MEP measurements within 24 h in patients with COPD. However, the outcomes of that study may have been influenced by other concurrent interventions, such as drug therapy for exacerbations and natural recovery from the worsening of symptoms (57). In addition, KT was effective in enhancing MIP when combined with inspiratory muscle training over a four-week period in healthy males. These findings suggest that KT may offer enhanced benefits when combined with other pulmonary rehabilitation methods (61). Moreover, in mechanically ventilated intensive care patients, KT contributed to delaying the rate of development of respiratory muscle atrophy and increased muscle thickness when applied to the thoracic wall (18).

Conversely, some studies have reported no significant improvement in respiratory muscle strength following KT application (17, 62). In healthy participants, applying KT to the diaphragm and accessory muscles had no impact on muscle strength (62). These adverse results may be attributed to the fact that the researchers only assessed the immediate effects of KT and did not incorporate spirometric or lung volume measurements in their evaluations. Moreover, KT application to the diaphragm did not improve respiratory muscle strength in patients with COPD (17). A possible reason for these findings is the unique structure of the diaphragm muscle. The KT muscle technique requires taping to follow the muscle’s anatomical and biomechanical features, such as its origin and insertion points, fiber orientation, and pinning angle. However, the distinctive shape, position, and movement of the diaphragm may have made it difficult to apply the taping precisely.

The findings of this study demonstrated a significant improvement in quality of life (QOL) in the KT group compared to the control group (*p* < 0.001). The observed enhancement in QOL scores, particularly in the domains of physical mobility and pain, can be attributed to the reduction in pain. Even a one-point decrease in pain can positively impact QOL by alleviating movement restrictions due to pain, reducing discomfort during coughing, and decreasing dependence on additional pain medication (19, 63). In a different population group, the minimal clinically important difference (MCID) for the SF-36 physical component summary (PCS) score was established as 4.1 (64). Consistent with the results of the current study, KT significantly enhanced the QOL of patients with mastectomy-associated lymphedema (52). Moreover, the integration of KT with resistance training proved to be more effective than exercise alone in improving the quality of life of breast cancer survivors (BCS) after mastectomy. Consequently, KT can be recommended as a non-invasive, supportive adjunct to standard rehabilitation programs for breast cancer survivors to facilitate healing (65).

In contrast, no statistically significant changes were detected in physical functioning, pain, overall health, energy levels, social interactions, emotional role limitations, or mental health in the KT group compared to baseline following thoracotomy (22). These findings could be attributed to the brief KT application period (1 week), absence of a placebo group, variations in the application technique, and absence of blinding of physiotherapists applying the KT, which could lead to biased outcomes. Correspondingly, KT did not yield additional gains in the quality of life of patients receiving non-steroidal anti-inflammatory drugs for post-thoracotomy pain syndrome (53). These findings may be explained by the short KT application period (only 5 days), significant baseline differences between the treatment groups in terms of pain duration, and variations in patient age and taping techniques.

The findings of this study indicate a positive correlation between muscle strength and quality of life in the Kinesio-taping group compared to the control group, aligning with the results of Demir et al. (66) and Pehlivan et al. (67). The strength of the respiratory muscles, particularly the maximal inspiratory pressure (MIP), in patients with atrial fibrillation was significantly correlated with the physical component score of the SF-36 (66). Moreover, the MIP value indirectly influences the quality of life of patients with idiopathic pulmonary fibrosis by affecting their exercise capacity (67). Additionally, both cardiorespiratory and muscular fitness are positively associated with health-related quality of life (HRQoL), especially in the domains of physical well-being, psychological health, and peer relationships (68). Similarly, research involving patients with heart failure identified a negative correlation between MIP and maximal expiratory pressure (MEP) and Minnesota Living with Heart Failure Questionnaire scores, suggesting that diminished respiratory muscle strength is associated with a poorer health-related quality of life (69). In contrast to the findings of this study, maximal inspiratory pressure (MIP) does not appear to limit health-related quality of life in healthy, physically active older adults (70), and this discrepancy may be attributed to differences in the populations under study.

### 4.1 Limitations

This study had several notable limitations. First, the lack of long-term follow-up restricts the evaluation of the lasting impact of Kinesio-taping on pain relief, pulmonary function, respiratory muscle strength, and quality of life beyond the four-week intervention period, limiting the understanding of its long-term benefits or potential adverse effects. Second, the study was conducted at a single hospital, which may have introduced selection bias and reduced sample diversity. Consequently, the findings may not be generalizable to broader or more varied populations, including patients from different geographic locations, healthcare settings, or cultural contexts. To validate and expand on these findings, future research should include multicenter trials with extended follow-up periods.

## 5 Conclusion

Based on the findings of this randomized controlled trial, integrating Kinesio-taping into standard chest physiotherapy for post-mastectomy patients should be considered a clinically effective intervention, as it significantly reduces pain and enhances pulmonary function, respiratory muscle strength, and overall quality of life compared with physiotherapy alone.

## 6 Clinical implementation

Integrating Kinesio-taping (KT) into the rehabilitation of mastectomy patients offers clinicians a range of benefits, including enhanced treatment outcomes, such as improved pain relief, pulmonary function, and respiratory muscle strength, leading to faster recovery and increased care effectiveness. KT also boosts patient satisfaction and adherence by providing visible relief and strengthening the clinician-patient relationship. As a non-invasive, low-risk, and cost-effective modality, KT expands the therapeutic toolkit and allows for personalized and holistic care. Additionally, gaining expertise in KT enhances professional credibility, supports conservative pain management, and may increase referrals and recognition in oncology rehabilitation settings.

To ensure the effective implementation of kinesio-taping after mastectomy, clinicians should receive specialized training in post-surgical taping techniques and patient assessment. KT should be integrated into individualized chest physiotherapy plans, considering each patient’s surgical site, pain level, and respiratory status. Regular monitoring of skin integrity and patient response is essential to ensure safety and effectiveness.

## Data Availability

The original contributions presented in the study are included in the article/supplementary material, further inquiries can be directed to the corresponding author.

## References

[1] Mehta J Vaghela N Patel H. The effect of physiotherapy in patients with modified radical mastectomy. Natl J Physiol Pharm Pharmacol. (2017):1. doi: 10.5455/njppp.2018.8.0829708082017

[2] Jiménez-Tornero J Cortés-Flores AO Chávez-Tostado M Morgan-Villela G Zuloaga-Fernández Del Valle C Zuloaga-Fernández Del Valle R . Effect of a preoperative single-dose steroid on pulmonary function and postoperative symptoms after modified radical mastectomy: results of a randomized clinical trial. Gland Surg. (2020) 9:1313–27. doi: 10.21037/gs-20-366, 33224806 PMC7667090

[3] Casla S López-Tarruella S Jerez Y Marquez-Rodas I Galvão DA Newton RU . Supervised physical exercise improves VO2max, quality of life, and health in early stage breast cancer patients: a randomized controlled trial. Breast Cancer Res Treat. (2015) 153:371–82. doi: 10.1007/s10549-015-3541-x, 26293147

[4] O'Donnell DE Webb KA Langer D Elbehairy AF Neder JA Dudgeon DJ. Respiratory factors contributing to exercise intolerance in breast cancer survivors: a case-control study. J Pain Symptom Manag. (2016) 52:54–63. doi: 10.1016/j.jpainsymman.2016.01.004, 26975626

[5] Yu AF Jones LW. Breast cancer treatment-associated cardiovascular toxicity and effects of exercise countermeasures. Cardiooncology. (2016) 2:1. doi: 10.1186/s40959-016-0011-5, 28133540 PMC5268817

[6] Odinets T Briskin Y Pityn M. Effect of individualized physical rehabilitation programs on respiratory function in women with post-mastectomy syndrome. Physiother Theor Pract. (2018) 35:419–26. doi: 10.1080/09593985.2018.1444117, 29482414

[7] Esmealy L Allahbakhshian A Gholizadeh L Khalili AF Sarbakhsh P. Effects of early mobilization on pulmonary parameters and complications post coronary artery bypass graft surgery. Appl Nurs Res. (2023) 69:151653. doi: 10.1016/j.apnr.2022.151653, 36635009

[8] Main E Denehy L. Cardiorespiratory physiotherapy: adults and paediatrics: first South Asia edition-E-book Elsevier Health Sciences. India: Elsevier (2017).

[9] Zeng R Tian K Xiao Z. Effectiveness of thoracic Kinesio-taping on respiratory function and muscle strength in patients with chronic obstructive pulmonary disease: a protocol of randomized, double-blind placebo-controlled trial. Medicine (Baltimore). (2021) 100:e25269-e. doi: 10.1097/MD.00000000000025269 33832089 PMC8036067

[10] Brateanu D. Kinesio-taping technique and kinesio tex. Timisoara Phys Educ Rehabil J. (2009) 2:36–40.

[11] Goksu H. Comparative efficacy of Kinesio-taping and local injection therapy in patients with subacromial impingement syndrome. Acta Orthop Traumatol Turc. (2015). 50:483–488. doi: 10.3944/AOTT.2015.15.0301 PMC6197412 27670388

[12] Thelen MD Dauber JA Stoneman PD. The clinical efficacy of kinesio tape for shoulder pain: a randomized, double-blinded, clinical trial. J Orthop Sports Phys Ther. (2008) 38:389–95. doi: 10.2519/jospt.2008.2791, 18591761

[13] Öztürk G Külcü DG Mesci N Şilte AD Aydog E. Efficacy of kinesio tape application on pain and muscle strength in patients with myofascial pain syndrome: a placebo-controlled trial. J Phys Ther Sci. (2016) 28:1074–9. doi: 10.1589/jpts.28.1074, 27190430 PMC4868190

[14] Kamper SJ Henschke N. Kinesio-taping for sports injuries. Br J Sports Med. (2013) 47:1128–9. doi: 10.1136/bjsports-2013-093027, 24159095

[15] Cho H-y Kim E-H Kim J Yoon YW. Kinesio-taping improves pain, range of motion, and proprioception in older patients with knee osteoarthritis. Am J Phys Med Rehabil. (2015) 94:192–200. doi: 10.1097/PHM.0000000000000148, 25706053

[16] Zanini M Nery RM de Lima JB Buhler RP da Silveira AD Stein R. Effects of different rehabilitation protocols in inpatient cardiac rehabilitation after coronary artery bypass graft surgery. J Cardiopulm Rehabil Prev. (2019) 39:E19–25. doi: 10.1097/HCR.0000000000000431, 31343586

[17] Tomruk M Keleş E Özalevli S Alpaydin AÖ. Effects of thoracic kinesio-taping on pulmonary function, respiratory muscle strength, and functional capacity in patients with chronic obstructive pulmonary disease: a randomised controlled trial. Explore. (2020) 16:332–8. doi: 10.1016/j.explore.2019.08.018, 31611155

[18] Metin Ökmen B Şengören Dikiş Ö Ökmen K Altan L Yildiz T. Investigation of the effect of kinesiotaping on the respiratory function and depression in male patients with chronic obstructive pulmonary disease: a prospective, randomized, controlled, and single-blind study. Aging Male. (2019) 23:648–54. doi: 10.1080/13685538.2019.1567703, 30739540

[19] Imperatori A Grande A Castiglioni M Gasperini L Faini A Spampatti S . Chest pain control with kinesiology taping after lobectomy for lung cancer: initial results of a randomized placebo-controlled study. Interact Cardiovasc Thorac Surg. (2016) 23:223–30. doi: 10.1093/icvts/ivw110, 27130717

[20] Jiandani MP Koradia C Mehta AA. Effects of kinesiotaping on pain and pulmonary function following open heart surgery: a randomized control trail. J Soc Indian Physiother. (2017) 1:36–41.

[21] Türk SG Çelik HK Çelik B Akça Z. Evaluation of the change in pain, dyspnea perception, and pulmonary function values with pain tape application in patients undergoing thoracotomy. Curr Thorac Surg. (2022) 7:14. doi: 10.26663/cts.2022.003

[22] Aksu NT Erdoğan M Erdoğan A. Effect of Kinesio-taping on pain, respiratory function, and muscle strength after thoracotomy. Turk J Thorac Cardiovasc Surg. (2023) 31:507–16. doi: 10.5606/tgkdc.dergisi.2023.24407, 38075992 PMC10704522

[23] Donec V Kubilius R. The effectiveness of Kinesio-taping(®) for mobility and functioning improvement in knee osteoarthritis: a randomized, double-blind, controlled trial. Clin Rehabil. (2020) 34:877–89. doi: 10.1177/0269215520916859, 32372651 PMC7376619

[24] Krajczy M Bogacz K Luniewski J Szczegielniak J. The influence of kinesio-taping on the effects of physiotherapy in patients after laparoscopic cholecystectomy. ScientificWorldJournal. (2012) 2012:948282:1–5. doi: 10.1100/2012/948282, 22645478 PMC3356750

[25] de Campos L Neves R Isoppo KS. Effects of Kinesio-taping® on pulmonary function of individuals with COPD: a systematic review and meta-analysis. Heart Lung. (2023) 57:236–42. doi: 10.1016/j.hrtlng.2022.09.021, 36272221

[26] Kang H. Sample size determination and power analysis using the G*power software. J Educ Eval Health Prof. (2021) 18:17. doi: 10.3352/jeehp.2021.18.17, 34325496 PMC8441096

[27] Kase K. Clinical therapeutic applications of the Kinesio (! R) taping method. 2nd ed. Albuquerque: Kinesio-taping Association, Dallas (2003). 12 p.

[28] Vilč B Šečić A Kirac I Herman I Kraljević N Brnić S. Guided imagery and music in the preoperative period and during radiotherapy in University Hospital for Tumors, Sestre milosrdnice University Hospital Center in Zagreb, Croatia. Lib Oncol. (2020) 47:78–83. doi: 10.20471/LO.2019.47.02-03.15

[29] Sciaky A Pawlik A Johanson M . Interventions for acute cardiopulmonary conditions In: Hillegass E, editor. Essentials of cardiopulmonary physical therapy. 4th ed. St Louis (MO): Elsevier (2017). 539–60.

[30] Main E Denehy L. Cardiorespiratory physiotherapy: adults and paediatrics: formerly physiotherapy for respiratory and cardiac problems Elsevier Health Sciences. Edinburgh: Elsevier. (2016).

[31] Pryor JA Prasad SA. Physiotherapy techniques In: Pryor JA Prasad SA, editors. Physiotherapy for respiratory and cardiac problems: adults and paediatrics. 4th ed. Edinburgh: Churchill Livingstone (2008). 136–63.

[32] Ahmad AM. Essentials of physiotherapy after thoracic surgery: what physiotherapists need to know. A narrative review. Korean J Thorac Cardiovasc Surg. (2018) 51:293–307. doi: 10.5090/kjtcs.2018.51.5.293, 30402388 PMC6200172

[33] Heuer A Rodriguez NE. Comprehensive respiratory therapy exam preparation guide Jones & Bartlett Learning. Burlington: Massachusetts (2017).

[34] Dean E Frownfelter DL. Cardiovascular and pulmonary physical therapy: evidence and practice. Mosby St. Louis, MO, USA: Elsevier Health Sciences (2006).

[35] Collins S Bradley N Fitzgibbon S McVeigh JG. Kinesiology taping for breast lymphoedema after breast cancer treatment: a feasibility randomised controlled trial. Physiother Pract Res. (2018) 39:107–16. doi: 10.3233/PPR-180113

[36] Delgado DA Lambert BS Boutris N McCulloch PC Robbins AB Moreno MR . Validation of digital visual analog scale pain scoring with a traditional paper-based visual analog scale in adults. JAAOS Glob Res Rev. (2018) 2:e088-e. doi: 10.5435/JAAOSGlobal-D-17-00088, 30211382 PMC6132313

[37] Society ATSER. ATS/ERS statement on respiratory muscle testing. Am J Respir Crit Care Med. (2002) 166:518–624. doi: 10.1164/rccm.166.4.518, 12186831

[38] Pellegrino R Viegi G Brusasco V Crapo RO Burgos F Casaburi R . Interpretative strategies for lung function tests. Eur Respir J. (2005) 26:948–68. doi: 10.1183/09031936.05.00035205, 16264058

[39] Miller MR Hankinson J Brusasco V Burgos F Casaburi R Coates A . Standardisation of spirometry. Eur Respir J. (2005) 26:319–38. doi: 10.1183/09031936.05.00034805, 16055882

[40] Sheikh KA Yagoub U Elsatouhy M Al Sanosi R Mohamud SA. Reliability and validity of the Arabic version of the SF-36 health survey questionnaire in population of khat chewers—Jazan region-Kingdom of Saudi Arabia. Appl Res Qual Life. (2013) 10:1–13. doi: 10.1007/s11482-013-9291-1

[41] Yánez-Brage I Pita-Fernández S Juffé-Stein A Martínez-González U Pértega-Díaz S Mauleón-García A. Respiratory physiotherapy and incidence of pulmonary complications in off-pump coronary artery bypass graft surgery: an observational follow-up study. BMC Pulm Med. (2009) 9:36. doi: 10.1186/1471-2466-9-36, 19638209 PMC2727489

[42] Riesenberg H Lübbe AS. In-patient rehabilitation of lung cancer patients—a prospective study. Support Care Cancer. (2009) 18:877–82. doi: 10.1007/s00520-009-0727-y, 19714371

[43] Canet J Gallart L Gomar C Paluzie G Vallès J Castillo J . Prediction of postoperative pulmonary complications in a population-based surgical cohort. Anesthesiology. (2010) 113:1338–50. doi: 10.1097/ALN.0b013e3181fc6e0a, 21045639

[44] Çınar HU Kefeli Çelik H Çelik B Doğan C. Is respiratory physiotherapy effective on pulmonary complications after lobectomy for lung cancer? Turk Gogus Kalp Damar Cerrahisi Derg. (2020) 28:638–47. doi: 10.5606/tgkdc.dergisi.2020.19693, 33403137 PMC7759043

[45] Zamani S Hagigat S Okhovatian F. Physical therapy approach in shoulder impairment along with lymphedemea after breast cancer surgery: a case study. J Clin Physiother Res. (2017) 2:145–51. doi: 10.22037/jcpr.v2i3.17098

[46] Chen W-C Hong W-H Huang TF Hsu H-C. Effects of kinesio-taping on the timing and ratio of vastus medialis obliquus and vastus lateralis muscle for person with patellofemoral pain. J Biomech. (2007) 40:S318. doi: 10.1016/s0021-9290(07)70314-7

[47] Kalichman L Vered E Volchek L. Relieving symptoms of meralgia paresthetica using kinesio-taping: a pilot study. Arch Phys Med Rehabil. (2010) 91:1137–9. doi: 10.1016/j.apmr.2010.03.013, 20537313

[48] McGlone F Reilly D. The cutaneous sensory system. Neurosci Biobehav Rev. (2010) 34:148–59. doi: 10.1016/j.neubiorev.2009.08.004, 19712693

[49] Lumpkin EA Caterina MJ. Mechanisms of sensory transduction in the skin. Nature. (2007) 445:858–65. doi: 10.1038/nature05662, 17314972

[50] Brockmann R Klein H-M. Pain-diminishing effects of Kinesio® taping after median sternotomy. Physiother Theor Pract. (2018) 34:433–41. doi: 10.1080/09593985.2017.1422205, 29308962

[51] Klein HM Brockmann R Assmann A. Pain-diminishing effect of Kinesio-taping in patients after sternotomy. J Cardiothorac Surg. (2015) 10:10. doi: 10.1186/1749-8090-10-s1-a76, 25618146

[52] Tantawy S Kamel D. Effect of kinesio-taping on pain post laporoscopic abdominal surgery: randomized controlled trial. IJTRR. (2015) 4:250. doi: 10.5455/ijtrr.00000098

[53] Kurt Saruhan H Toprak M. Pain-diminishing and quality of life-related outcomes of Kinesio-taping in patients on non-steroidal anti-inflammatory drug therapy for post-thoracotomy pain syndrome. Turk J Phys Med Rehabil. (2020) 66:147–53. doi: 10.5606/tftrd.2020.4068, 32760891 PMC7401681

[54] Malehorn K Hiniker J Mackey T Heumann KJ Murray S Pettitt RW. Kinesio tape® applied to the thorax augments ventilatory efficiency during heavy exercise. Int J Exerc Sci. (2013) 6:157–63. doi: 10.70252/ISHK7260

[55] Arslan SA Daşkapan AD Pekyavaş NÖ Sakızlı E. Effects of kinesio-taping applied to diaphragm muscle on aerobic exercise capacity and pulmonary function in sedentary individuals. Anadolu Kliniği Tıp Bilimleri Dergisi. (2018) 23:68–72. doi: 10.21673/anadoluklin.385414

[56] Ganesh B Praneetha N Naik V. Comparison of two thoracic kinesio-taping techniques on pulmonary function, oxygen saturation, and exercise capacity in COPD patients: a randomized clinical trial. Indian J Phys Therapy Res. (2023) 5:32–6. doi: 10.4103/ijptr.ijptr_168_22

[57] Daitx RB dos Santos K Dohnert MB da Silva TA Silva J. Limited utility of Kinesio-taping® in the physiotherapy treatment for patients with chronic obstructive pulmonary disease exacerbation. Physiother Theor Pract. (2018) 34:741–6. doi: 10.1080/09593985.2018.1423658, 29308939

[58] Kalron A Bar-Sela S. A systematic review of the effectiveness of Kinesio-taping--fact or fashion. Eur J Phys Rehabil Med. (2013) 49:699–709. 23558699

[59] Fu T-C Wong AMK Pei Y-C Wu KP Chou S-W Lin Y-C. Effect of Kinesio-taping on muscle strength in athletes—a pilot study. J Sci Med Sport. (2008) 11:198–201. doi: 10.1016/j.jsams.2007.02.011, 17588814

[60] Słupik A Dwornik M Białoszewski D Zych E. Effect of Kinesio-taping on bioelectrical activity of vastus medialis muscle. Preliminary report. Ortop Traumatol Rehabil. (2007) 9:644–51. 18227756

[61] Lee M Kim M Ahn C. Impact of concurrent inspiratory muscle training and tape on inspiratory muscle strength, endurance and pulmonary function. J Korean Soc Integr Med. (2014) 2:65–73. doi: 10.15268/ksim.2014.2.3.065

[62] Zübeyir S Nilüfer K Burcu C Onur A Bahar K Ufuk YS . The effect of kinesiology taping on respiratory muscle strength. J Phys Ther Sci. (2012) 24:241–4. doi: 10.1589/jpts.24.241

[63] Fiorelli A Mazzella A Passavanti B Sansone P Chiodini P Iannotti M . Is pre-emptive administration of ketamine a significant adjunction to intravenous morphine analgesia for controlling postoperative pain? A randomized, double-blind, placebo-controlled clinical trial. Interact Cardiovasc Thorac Surg. (2015) 21:284–90. doi: 10.1093/icvts/ivv154, 26071592

[64] Carreon LY Glassman SD Campbell MJ Anderson PA. Neck disability index, short form-36 physical component summary, and pain scales for neck and arm pain: the minimum clinically important difference and substantial clinical benefit after cervical spine fusion. Spine J. (2010) 10:469–74. doi: 10.1016/j.spinee.2010.02.007, 20359958

[65] Ramadan AM ElDeeb AM Ramadan AA Aleshmawy DM. Effect of combined Kinesiotaping and resistive exercise on muscle strength and quality of life in breast cancer survivors: a randomized clinical trial. J Egypt Natl Canc Inst. (2024) 36:1. doi: 10.1186/s43046-023-00205-z, 38221574 PMC13313848

[66] Demir R Zeren M Gurses HN Yigit Z. Relationship of respiratory muscle strength, pulmonary function, and functional capacity with quality of life in patients with atrial fibrillation. J Int Med Res. (2018) 46:195–203. doi: 10.1177/0300060517723252, 28789604 PMC6011306

[67] Pehlivan E Zeren M Niksarlıoğlu EY Karaahmetoğlu FS Özcan ZB Balcı A . Investigation of respiratory muscle strength and its influence on exercise capacity and quality of life in patients with idiopathic pulmonary fibrosis. Sarcoidosis Vasc Diffuse Lung Dis. (2024) 41:e2024028. doi: 10.36141/svdld.v41i2.14884, 38940715 PMC11275540

[68] Bermejo-Cantarero A Álvarez-Bueno C Martínez-Vizcaino V Redondo-Tébar A Pozuelo-Carrascosa DP Sánchez-López M. Relationship between both cardiorespiratory and muscular fitness and health-related quality of life in children and adolescents: a systematic review and meta-analysis of observational studies. Health Qual Life Outcomes. (2021) 19:127. doi: 10.1186/s12955-021-01766-0, 33882937 PMC8059195

[69] de Noronha IM Almeida LX de Souza Silva Andra NV de França EET de Morais Lima JH Pedrosa R . Respiratory muscle strength and quality of life in patients with heart failure and their main correlated factors. J Cardiovasc Nurs. (2023) 39:535–42. doi: 10.1097/jcn.0000000000001062 37955376

[70] Roldán A Monteagudo P Cordellat A Sanchis-Soler G Blasco-Lafarga C. Inspiratory muscle strength and cardiorespiratory fitness association with health-related quality of life in healthy older adults. Front Sports Act Living. (2021) 3:624947. doi: 10.3389/fspor.2021.624947, 33817635 PMC8012766

